# On Quinidine in Intermittent Fever

**Published:** 1854-12

**Authors:** George L. Upshur

**Affiliations:** Surgeon to the U. S. Marine Hospital, Norfolk, Va., &c., &c.


*On Quinidine in Intermittent Fever.* By GEORGE L. UPSHUR, A. M., M. D. Surgeon to the U. S. Marine Hospital, Norfolk, Va., &c., &c.

Since the publication of Dr. Pepper's article, in the September number of this Journal, on the "*Treatment of Intermittent Fever by Quinidine*," I have given this agent a very fair trial in this class of diseases, of which I have seen a large number of cases, in hospital and private practice. Below is the result of the treatment in *thirty* cases,—twenty of which occurred in private practice, and the rest were treated in the hospital.

CASE 1st.—E. T., a female, aged 14, was seized with chill on the 21st of September, followed by high fever and intense aching of the head, back and limbs. The paroxysm came on at 8 o'clock, A. M. and was repeated on the 22d at the same hour. Visited her in the afternoon; pulse full, 120; skin dry and hot; thirst urgent; nausea and vomiting; tongue clean; bowels regular; no abdominal tenderness. Ordered Quinidine Sulph. gr. x., pill v.; one every hour.

23d.—Did not retain the two first pills; slept tolerably well, the fever declining about 5 o'clock, P. M., with perspiration; bowels moved once; no appetite; pulse 80, small; thirsty, and looks as if she would have another paroxysm. ℞. Quinidine Sulph. gr. x., chart. 5; one powder every hour. Two of the powders were thrown up as soon as swallowed; she missed the paroxysm, however, and has had no return. The whole quantity of quinidine retained was 16 grains.

CASE 2d.—J. G., æt. 17, was seized, September 21st, with quotidian intermittent, the paroxysm occurring at 10 A. M. Saw her on the evening of the 22d; skin hot and dry; pulse 136, small and feble; bowels regular, less aching than in the morning. ℞. Quinidine Sulph., gr. x.; to be given at one dose. The paroxysm recurred next day at the usual hour, but with some abatement in intensity. ℞. Quinidine Sulph. gr. x.; pill 5. One every hour. She missed the paroxysm on the next day, and had no return afterwards. There was slight ringing in the ears.

CASE 3d.—E. T. B., male, æt. 45, seized with intermittent of tertian type; the paroxysm occurring at 1 P. M. Saw him on 23d, and prescribed Quinidine Sulph gr. xv.; pill 4; one every hour. On the next day he had a light attack at the usual hour, and on the day after, (25th,) I ordered for him, Quinidine Sulph. gr. v.; pill 2; one every 3 hours. He had no return of the disease.

Case 4th.—R. F., a male, æt. 6 months, was seized on 22d of September, with tertian intermittent, the paroxysm occurring at 3 o'clock A. M. He has cough of several weeks standing, and is a good deal emaciated from cholera infantum; his fevers decline without moisture. I saw him on 25th, and prescribed, Quinidine gr. viii., aquæ ℥i. M. S. a teaspoonful every hour. The paroxysm came on next day as usual, but it did not return afterwards.

Case 5th.—T. F., a male, æt. 6 years, in the same house, seized Sept. 17, with intermittent of the quotidian type, the paroxysms occurring in the afternoon. The fever is usually very high, and declines without moisture—bowels regular—and appetite good. This child had recently had an attack of scarlatina. I saw him on 25th Sept., and ordered Quinidine gr. x. Pil. 5, one every hour. He had no return of the disease.

Case 6th.—J. C., male, æt. 6, seized Sept. 26th, with well marked chill, having for several days before, at the same hour, suffered from slight feverishness. On the 26th, the paroxysm came on in the night, the fever was high, and declined without moisture. I found him on the next day with a dry skin, and thirst, but no febrile movement; bowels were regular, and there was no abdominal tenderness. Ordered Quinidine gr. xii. Pil. 6, one every hour. He took all the pills, and had no return of the disease.

Case 7th.—Mrs. W., æt. 19, seized Sept. 23d., with tertian intermittent. The paroxysm comes on at noon, and is marked by long chill, intense fever, which declines without moisture, and general aching of the head, back, and limbs. She is nursing a very hearty child, and has a chronic diarrhœa of two months, the dejections being liquid, and yellowish, and unaccompanied by pain. Saw her Sept. 28th, during the paroxysm, and ordered Quinidine gr. xv., Pil. 6, one every hour. On the 30th, she had another paroxysm, but less intense, and I prescribed,

Quinidine gr. x. Pil. 5, one every two hours. She had no return of the disease.

Case 8th.—Mr. B., æt. 30, seized Sept. 25th with general aching, followed by fever; bowels became disturbed on 26th, the dejections being very frequent, liquid, and painless; on 27th he had nausea and vomiting; paroxysm of fever occurs every afternoon, being preceded by only a little stretching. Saw him on 28th: no abdominal pain, but the bowels are moved every hour; no appetite; skin dry; very thirsty; ℞. Quinidine gr. xv., acid sulph. dilut. ʒijss., tr. lav. c. ʒij., tr. opii ʒj., aquæ camph. q. s. ℥ii. M. A teaspoonful every hour. His bowels soon became relieved, and on the next day he was sitting up, missed the paroxysm, and had no return afterwards.

Case 9th.—Mrs. S. æt. 18, pregnant, seized 14th Sept, with quotidian intermittent. I saw her on the 28th. Up to the 25th, she had a well marked chill, followed by fever. Since the 25th, the paroxysm was ushered in by simple aching and thirst. She has been freely purged with calomel and jalap; spleen enlarged, no abdominal tenderness. ℞. Quinidine gr. xv.; Pil. 6, one every hour. She had no return of the disease.

Case 10th.—Miss S., æt. 16, was seized Sept. 30th, at 8 A. M., with chill followed by fever, which declined without moisture. On the 28th, she had slight chilliness, and aching, but the paroxysm was not well marked. Saw her Oct. 1st, and prescribed, Quinidine gr. xv., Pil. 6, one every hour. The last pill was taken at 9½ P. M. The paroxysm came on next day, Oct. 2d, an hour earlier, and was more intense; the back, head and limbs ached violently, pulse was 124, and full, with hot skin, and urgent thirst. The bowels not having been moved for two days, I ordered Pil. comp. cath., 3 to be taken at once. She missed the paroxysm on the 4th, and had no return of it.

Case 11th.—Mrs. B., æt. 33, seized Sept. 30th, with chill, well marked; thinks she had slight chills for several days before; tertain type. Saw her Oct. 2d; the paroxysm came on at 12 M. on 30th, and at 8 A. M. to-day; fever prolonged and declined with a dry skin; bowels regular; aching intense; nausea, and abdominal tenderness. ℞. Quinidine gr. x.; Pil. 5, one every hour. She missed the paroxysm on 4th, and had no return of the disease.

Case 12th.—Leah, colored, æt. 15, was seized with intermittent of tertian type, Sept. 30th. Saw her on 2d Oct.; the paroxysm occurred on that day at 11 A. M., and was characterized by the usual symptoms; bowels regular; no abdominal tenderness. Prescribed Quinidine gr. xv.; Pil. 6, one every hour. On the 4th, there was another paroxysm, more intense than any previous one. The fever continued unabated for upward of six hours, and, in addition to the intense aching of the head and limbs, she had violent pain at the epigastrium, with nausea and vomiting; bowels had not been moved for two days. Being absent from the city, the case was seen by my friend Dr. Robt. B. Tunstall, who prescribed calomel gr. x., to be taken at once, with a tablespoonful of the following mixture every hour, until the colic was relieved: ℞. Anodyn. Hoff. ℥ss. tr. lav. c. ℨss. aquæ q s. ℥iv. M. On the next day, Oct. 5th, I found the bowels had been freely moved, and the pain in the stomach entirely relieved. Ordered Quinidine gr. xv. Pil. 6, one every hour. She missed the paroxysm on 6th, and had no return of the disease.

Case 13th.—Was called to see S. W., male, æt. 9 years, on 1st of Oct. Has had quotidian intermittent for 30 days, the paroxysms occurring, with great regularity, at noon, and being remarkably severe in their character. For 25 days he was the victim of homœopathic nonsense, but, of course, without the slightest benefit. Every day the disciple of Hahnemann would give his infinitessimal powder an extra trituration, and, placing it upon the tongue of his dupe, gravely promise that he should not have a return of the chill. Failing to visit the patient on the 26th day, he was sent for, and returned for answer, that "really it would give him great pleasure to *cure* the case, but he was *too busy* to attend it longer."

I found the patient a good deal emaciated, very sallow, and unable to leave his bed. Skin dry, tongue foul, spleen excessively enlarged, and abdomen tumid. Ordered Quinidine gr. xvi. Pil. 8, one every two hours. Visited him next day at 5 P. M., he had taken but six pills, but the paroxysm had not returned, and he was more comfortable than he had been for a month past; ordered him to take the two remaining pills, and, as he was decidedly anemic, gave the following; ℞. Quinidine gr. xv., Ferri per Hydrog. ℈i., Ext. Gent. ℈ij., M. Pil. 20, S. one pill three times a day. He had no return of the disease.

CASE 14th.—S. B., male, æt. 18, was seized with intermittent Sept. 26th, visited him Oct. 2d. Has a paroxysm in the morning one day, and in the afternoon next day; double tertian type. The paroxysms are ushered in by slight chilliness, and attended by intense aching, and abdominal tenderness; bowels regular, and fever declines with a dry skin. ℞. Quinidine gr. x. Pil. 5, one every hour. On the 4th, I made this note: had no chill yesterday, but fever with aching, &c. This morning, feels languid, but has no fever, and no aching; complexion very sallow; tongue clean and pale; no appetite; ℞. Quinidine gr. x.; Pil. 5, one every hour. At my visit on 6th, found him walking about, and fairly convalescent. He had no relapse.

CASE 15th.—I. T., male, æt. 10, seized Sept. 22d, quotidian type; chills not well marked; febrile movement considerable, accompanied by thirst, aching, &c., and declines with a dry skin; the paroxysm is worse every other day; bowels regular; some abdominal tenderness. I visited him Sept. 26th, and prescribed, Quinidine gr. xv., Pil. 6, one every hour. On 27th and 28th, the paroxysms came on as usual, with no abatement in their severity. ℞. Quinidine gr. xv., Pil. 6, one every two hours. Their being no improvement on the 1st of October, I ordered, *Quiniæ Sulph.* gr. xx., Pil. 8, one every two hours. He missed the paroxysm on the 3d, and had no return afterwards.

CASE 16th.—Mrs. H., æt. 36, sent for me Sept. 29th. For eight days before, she had suffered from headache and fever, which came on every day at 11 A. M., lasted about six hours, and declined without moisture. She has suffered very severely for two years past with frequent attacks of uterine hemorrhage, and is pale and enfeebled. Prescribed Quinidine gr. xv., Pil. 6, one every two hours.

Oct. 2d. Has had a paroxysm every day since the last note: cannot say they have been less severe. ℞. Quinidine gr. x., Pil. 5, one every hour.

Oct. 4th. In statu quo; the occurrence of the paroxysm seems so far to be uninfluenced by the treatment. ℞. *Quiniæ Sulph.* gr. xv., Pil. 6, one every two hours. She missed the paroxysm next day, and had no return of it.

Case 17th.—Mr. F., æt. 36, seized Oct. 1st, with quotidian intermittent; fever high, and aching severe; bowels confined. Prescribed for him on 3d, Quinidine gr. xv., Pil. 6, one every hour, and 3 Pil. c. c., at bedtime. He had a slight paroxysm on the next day, but no return afterwards.

Case 18th.—Mrs. M. æt. 50, seized Sept. 28th with slight chilliness, followed by fever; quotidian type; the paroxysm occurring rather irregularly as regards the hour; fever declines with a dry skin; during the attack there is violent aching of the head, back, and limbs, with nausea, vomiting and abdominal pain. Visited her on 2d Oct., and prescribed Quinidine gr. x., Pil. 5, one every hour. This did not stop the paroxysms, although they were much lighter. On 4th of Oct., I repeated the first prescription, and she had no return of the disease afterwards.

Case 19th.—Miss M., æt. 22, daughter of the above, was seized Sept. 24th, with quotidian intermittent. Saw her Oct. 2d. At first there was well marked chill, but for the last four days, the paroxysm has been ushered in by fever and aching only, and occurs with great regularity every day at noon. ℞. Quinidine gr. x., Pil. 5, one every hour. She did not begin to take the pills until late the next day, and the attack came on as usual. The day after, Oct. 4th, the paroxysm was very light, but on the 5th, it was as severe as ever. On the 6th, I repeated the first prescription, and she had no return of the disease.

Case 20th.—Mrs. S., æt. 50, seized Oct. 2d, early in the night, with slight chilliness, followed by high fever, and intense general aching. During the height of the paroxysm, she vomited a good deal of bilious matter, and was delirious. Saw her on the morning of the 3d, the fever had somewhat abated, but she was still much distressed; pulse 120, and very small; skin dry; face flushed, pain at the epigastrium; violent headache, with a disposition, now and then, to talk incoherently; thirst urgent; tongue foul; bowels confined; R. Quinidine gr. xv., mass. hyd. gr. x, pulv. rhei. gr. xv. M. Pil. 6. One every hour.

The second paroxysm came on the same evening at 8 o'clock with scarcely any abatement in intensity, and when I visited her on the 4th, I found her in nearly the same condition as at my first visit. R. Quinidine gr. xv. hyd. submur. gr. x. M. Pil. 6, one every hour. The next paroxysm came on at the same hour, but was much lighter, and, on the morning of the 5th, I found her nearly free from fever; pulse 84; skin perspirable; less aching of the head and limbs, and little or no nausea; ℞. Quinidine gr. x., Pil. 5, one every hour. From this time she had no return of the disease, and convalesced rapidly. There was no ringing in the ears.

Case 21st.—Walter J. Sault, æt. 19, was admitted into the hospital Sept. 12th. He had been trading through the Dismal Swamp canal, and, for four weeks, had suffered from intermittent fever, quotidian type, with a respite, now and then, of two or three days only. Saw him on 13th; the paroxysm comes on at 2 P. M., being ushered in by well marked chill, and declining with perspiration; bowels regular; tongue nearly clean; appetite impaired; complexion sallow; abdomen tender on pressure; and spleen much enlarged; ℞. Quinidine gr. x. Pil. 5, one every hour. He commenced taking the pills during the height of the fever.

The paroxysm came on next day at 7 P. M., being a postponement of five hours; there was no abatement in its severity. Ordered Quinidine gr. x., Pil. 5, to be taken as before. He remained in the hospital until the 22d, and had no return of the disease. A week after he was discharged, he came to my office, to say, that the chills had re-appeared, having had an attack the day before. I prescribed the Quinidine as in the first instance, and up to this time, (Oct. 20th,) he has not relapsed.

Case 22d.—Charles Scott, æt. 19, was admitted Sept. 14th. He had been trading up James River, and on the 4th, was seized with quotidian intermittent, the paroxysms occurring every afternoon. Visited him at 7 P. M., skin very moist; pulse 100; tongue clean; bowels open; thirsty, no abdominal tenderness. ℞. Quinidine gr. x. Pil. 5, one every hour. He took all the pills by 10 o'clock next morning, but the paroxysm came on at 4 P. M., with unabated severity. 16th, visit paid at 1 P. M., complains of pain in the abdomen; bowels moved once to-day; pallor and headache, and is evidently going to have another paroxysm. ℞. Quinidine gr. x., Pil. 5, one every hour, beginning at daybreak to-morrow. 17th, visited him at 3 P. M.; paroxysm came on yesterday at 5 P. M.; a postponement of one hour; feels better to-day than usual; no medicine. 18th, had a very slight attack yesterday at 6 P. M., it lasted only an hour, and the aching was scarcely perceptible. ℞. Quinidine gr. x. Pil. 5, one every hour. 19th. He missed the paroxysm yesterday, but is pale, feeble, and incapable of exertion. ℞. Ferri Sulph. gr. xx., ext. gent. ʒi. M. Pil. 20, one pill 3 times a day. He had no return of intermittent while he remained in the house, which was until the 22d Sept.

Case 23d.—Alexander, æt. 30, entered Sept. 19th. He had been trading to Plymouth, N. C., and was seized on the 5th with quotidian intermittent, characterized by the usual symptoms. The paroxysm comes on in the afternoon, being preceded by only slight chilliness, thirst, &c.; the fever lasts nearly all night, and declines with a moist skin; tongue clean; bowels regular; ℞. Quinidine gr. x. pil. 5, one every hour. The paroxysm on the next day was very slight, and of less than half the ordinary length. He still remains (Nov. 10th) in the hospital with an attack of syphilis, but has had no return of the intermittent.

Case 24th.—Rooks, æt. 25, entered Sept. 19th. Has been trading up the James river, where miasmatic fever is very prevalent at this season. Seized on the 14th, at night, with general aching, followed by high fever; on 16th, had a well marked chill, and another on 18th, tertian type; anorexia; tongue clean and pale; headache; no abdominal pain; bowels constipated; thirst; moist skin; ℞. Quinidine gr. x. mass. hyd., pulv. rhei. *aa.* gr. xii., M. pil. 5. one pill every hour. On the 20th, he had another paroxysm, and another on 22d. On this day, I repeated the quinidine, gr. x. pil 5, one every hour. He missed the paroxysm on the 24th, and left the house on the 25th. This man was pretty severely ptyalized by 12 grains of blue mass.

Case 25th.—Parker, æt. 43, previously healthy, entered Sept. 22d, was seized on the 18th with chill, followed by fever and sweating; has had a paroxym every day since, occurring at 9, A. M. On the day he entered, his fever was not preceded by chill; aching not very intense; thirst; tongue clean; appetite tolerably good; bowels confined; spleen a good deal enlarged; ordered pil. c. c. 3 at once. ℞. Quinidine gr. x. pil. 2; one at 5 o'clock, and the other at 9, P. M.

Sept. 23d.—Has not missed the paroxysm, although it was postponed two hours; fever more decided, and more distressing, accompanied by nausea and vomiting; pulse 120, full and resisting; thirst urgent; bowels loose; ℞. Quinidine gr. x. pil. 5; one every hour. He missed the attack next day, but it returned on 25th in the afternoon, with its usual intensity; ℞. Quinidine gr. x. pil. 4, one every two hours. On the 26th, he had another paroxysm, and having already taken 30 grains of *quinidine*, without a favorable result, I gave him the following: ℞. Quiniæ. sulph. gr. xx. chart. 4; one every two hours. He had no return of the disease, and was discharged on 30th of October; having remained until this time, to be treated for enlarged spleen and general debility, the result of the fever.

Case 26th.—Ballestry, æt. 21, entered Sept. 25th. Has been trading up James river, and was seized on 20th with chill, fever and general aching; tertian type; thirsty; tongue clammy and foul; pulse 120; bowels regular; no abdominal uneasiness; paroxysm occurs in the morning; ℞. Quinidine gr. xv. pil. 6, one every hour. He missed the attack on the 26th, and left the house next day.

Case 27th.—Mathias, æt. 27, entered Sept. 26th; been trading through the Dismal Swamp Canal; seized on 23d with chill, followed by fever, which declined with a moist skin; tertian type; had a paroxysm yesterday at 5, P. M.; bowels open; skin moist; appetite impaired; nausea and vomiting; ℞. Quinidine gr. xv., pil. 6, one every hour. He had no return, and was discharged on 30th.

Case 28th.—Flynn, æt. 22, stout and previously healthy, entered Sept. 30th. Is last from Savannah, where he remained one month. Seized on 28th, at 11 o'clock, A. M., with chill, followed by fever, &c. Has had a paroxysm every day since, the one to-day coming on at 8 o'clock, A. M. The chilliness is very slight, but the fever and aching are intense; bowels regular; tongue foul; skin moist; thirst; anorexia; no abdominal tenderness; ℞. Quinidine gr. xv., pil. 6, one every hour. He was discharged Oct. 12th, having had no return of the disease.

Case 29th.—Gibson, æt. 32, stout and previously healthy, entered Sept. 30th. He is from the same vessel as the preceding; seized on 25th with pain in the head and back, without chill; but high fever, which declined with sweating; has had a similar paroxysm every day since, his chief distress being in the head; bowels regular; tongue foul and dry; urgent thirst; abdominal tenderness; no nausea; ℞. Quinidine gr. xv., pil. 6, one every hour.

Oct. 1st.—Passed a very bad night; restless and delirious; paroxysm came on this morning at 8 o'clock; *less intense* than usual; no thirst; head and back ache violently; nausea and vomiting; pulse 96, and soft; tongue dry and fissured; ℞. Quinidine gr. x., pil. 5, one every hour.

On the next day, (2d,) he had the paroxysm as usual, and all of his symptoms were aggravated; the tongue being dry and cracked; eyes injected; skin yellow; bowels confined, and mind disposed to wander; ℞. Quinidine sulph. gr. xx., mass. hyd. gr. xv., pulv. rhei. gr. x., M. pil. 8, one every two hours.

Oct. 3d.—Free from fever; skin moist; head ache nearly disappeared; bowels still unmoved; ℞. pulv. jalap. comp. ʒiss, to be given at once. I found him fairly convalescing on the 4th, and he was discharged cured on the 11th.

Case 30th.—Mozart, æt. 34, previously healthy, entered Oct. 3d. Has been trading up James river, and was seized about 1st of September with quotidian intermittent, the paroxysms occurring at 2½ o'clock, P.M. Ten days ago the disease assumed the tertian type; chills are decided; fever high, aching intense; had a paroxysm yesterday; sweats freely; has taken the sulphate of quinia several times in considerable doses, with the effect of putting a stop to the attacks for a few days; bowels regular; a good deal of nausea; no abdominal pain; tongue clean; conjunctiva yellow. ℞. Quinidine gr. xv., pil. 6, one pill every hour.

Oct. 4th.—Had a paroxysm last night between 12 and 1 o'clock; lighter than usual; feels better to day; some tinnitus aurium; no medicine. On the 6th the paroxysm was characterised by merely a little malaise. I repeated the quinidine in the same dose, and he had no return while he remained in the house, which was until the 9th of October.

This man was re-admitted on 27th of October with a second attack of quotidian intermittent of a week's duration. He took 15 grs. of quinidine as in the first instance, and was discharged, cured, on the 1st of November.

These cases are sufficient to show that quinidine is undoubtedly an agent of considerable efficacy in the treatment of intermittent fever. I am not yet prepared, however, to assent fully to Dr. Pepper's remark, that the quinidine is more active than either sulphate of quinia or cinchonia. A large majority of my cases required from fifteen to thirty grains to arrest the paroxysm, while in several, after the fairest trial, the disease did not succumb until *quinine* was resorted to. If this communication were not already too long, it would be interesting to institute a comparison between the power of quinine and quinidine, by detailing in this connection a few cases treated by the former remedy. Enough has been said, however, to show that the subject is one of great practical interest, and well worthy the attention of those who have ample opportunities of observation in this class of diseases.

During the past season I treated a few cases of remittent, and the graver forms of miasmatic fever with quinidine. The results were exceedingly satisfactory, and I have no hesitation in saying that as an anti-miasmatic it holds in my confidence the next place to sulphate of quinia.

The article which I used was obtained from Powers & Weightman, by my excellent friend, R. S. Bernard, druggist, of this city.

Should I live, I propose, during another season, to pursue this investigation, and, in the meantime, I commend the subject to the attention of medical men in other localities.

*Norfolk, Va., Nov. 10th,* 1854.

